# Endovascular Repair with a Stent Graft in a Patient with Aortoduodenal Fistula after Radiation Therapy

**DOI:** 10.1155/2017/2087142

**Published:** 2017-10-17

**Authors:** Kazuhiko Morikawa, Hirokazu Ashida, Yosuke Nozawa, Kenji Motohashi, Takao Igarashi, Hiroya Ojiri, Yuji Kanaoka, Takao Ohki

**Affiliations:** ^1^Department of Radiology, The Jikei University School of Medicine, Tokyo, Japan; ^2^Department of Vascular Surgery, The Jikei University School of Medicine, Tokyo, Japan

## Abstract

Primary aortoduodenal fistula (ADF) is a direct communication between the abdominal aorta and the gastrointestinal tract without any previous vascular intervention and represents a rare but critical cause of repeated and massive gastrointestinal bleeding. Primary ADF often occurs as a result of atherosclerotic aneurysm and infection, but ADF involving a normal-size aorta is rare; furthermore, ADF related to radiation therapy is extremely rare. We present the case of a 56-year-old man with a history of bowel obstruction due to radiation enteritis who was admitted with severe hematemesis and hemorrhagic shock. Gastroduodenal endoscopy and contrast-enhanced computed tomography findings were unremarkable. Aortoduodenal fistula was suspected based on the diffuse calcification of the abdominal aorta confined to the radiation field and the presence of an aortoduodenal communication on angiography. Endovascular repair with a stent graft seemed to be a safer option than open surgery and was suited to the rapid control of bleeding from ADF because of the patients' unstable hemodynamic state and the presence of intestinal adhesions. The fistula was successfully sealed by endovascular stent graft placement. Hematemesis did not recur postoperatively and anemia gradually improved. The patient died from pneumonia 33 days later.

## 1. Introduction

Aortoduodenal fistula (ADF) is a direct communication between the abdominal aorta and the gastrointestinal tract and represents a rare but critical cause of repeated and massive gastrointestinal bleeding. Therefore, accurate diagnosis and immediate treatment are necessary. AEF is classified into the following two types: primary and secondary. Most commonly, ADF occurs from surgical repair of a prior aortic aneurysm (secondary ADF); however, ADF can arise due to the spontaneous development of a connection between the duodenum and the abdominal aorta (primary ADF) [1]. Primary ADF often occurs as a result of atherosclerotic aneurysm and infection [1]. ADF involving a normal-size aorta is rare [2]; furthermore, ADF related to radiation therapy is extremely rare. Herein, we present a case of ADF related to radiation-induced arteriosclerosis/aortopathy and enteritis, treated with an endovascular stent graft.

## 2. Case Presentation

**Figure 1:**
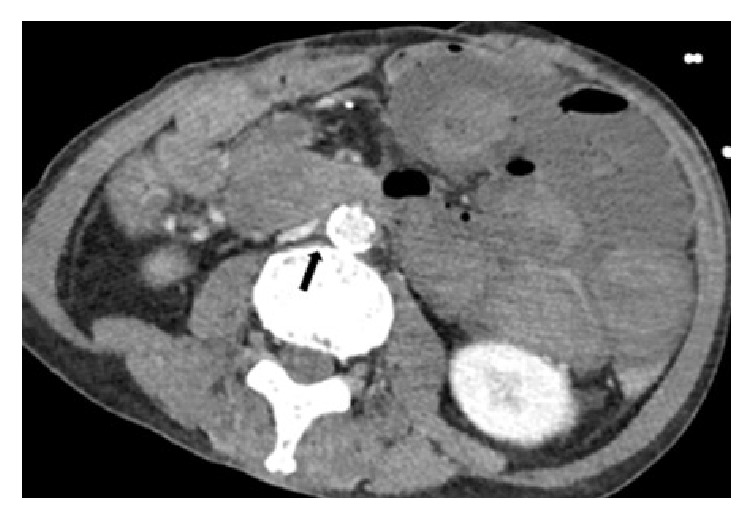
Contrast-enhanced computed tomography image on admission. A massive hematoma is visible in the small intestine, with mucosal thickening of the duodenal wall, duodenal stenosis at the level of the third and fourth portions of the duodenum, and soft-tissue density (arrow) with fat stranding between the duodenum and aorta.

**Figure 2:**
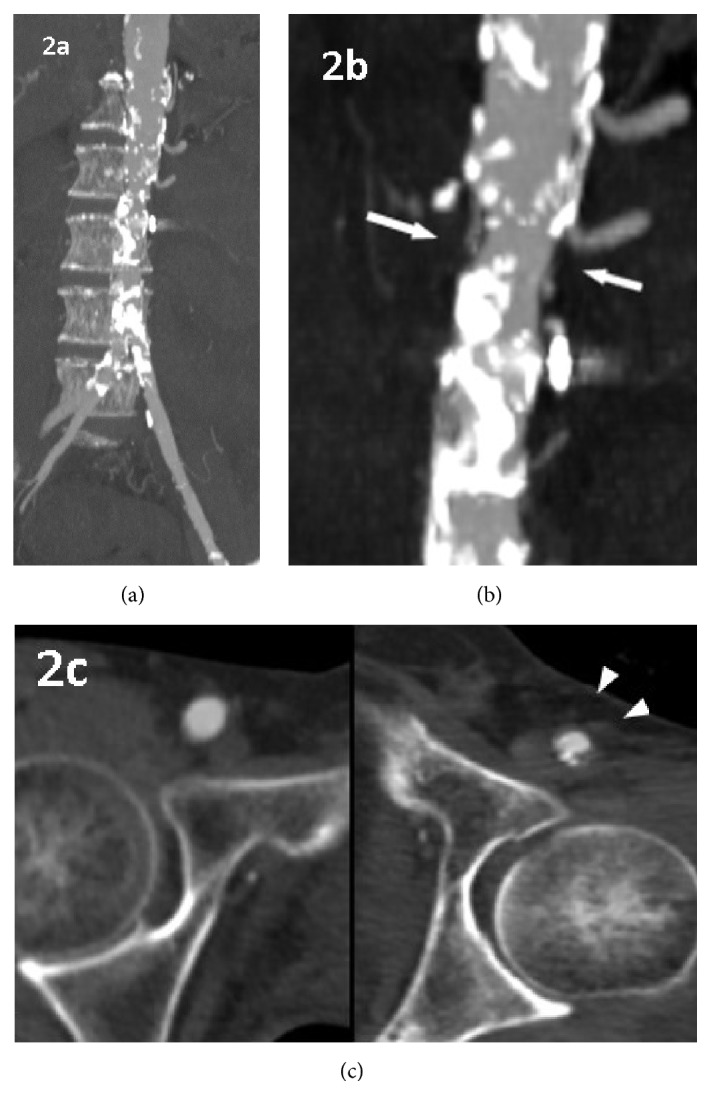
Maximum intensity projection reconstruction of coronal computed tomography angiogram and transverse plane at the level of inguinal region on admission. (a) Diffuse calcification from the abdominal aorta to the left common iliac artery was observed, which was confined to the irradiation field. ((b) and (c)) Irregular stenosis of the abdominal aorta (white arrows) and left femoral artery (arrowhead).

**Figure 3:**
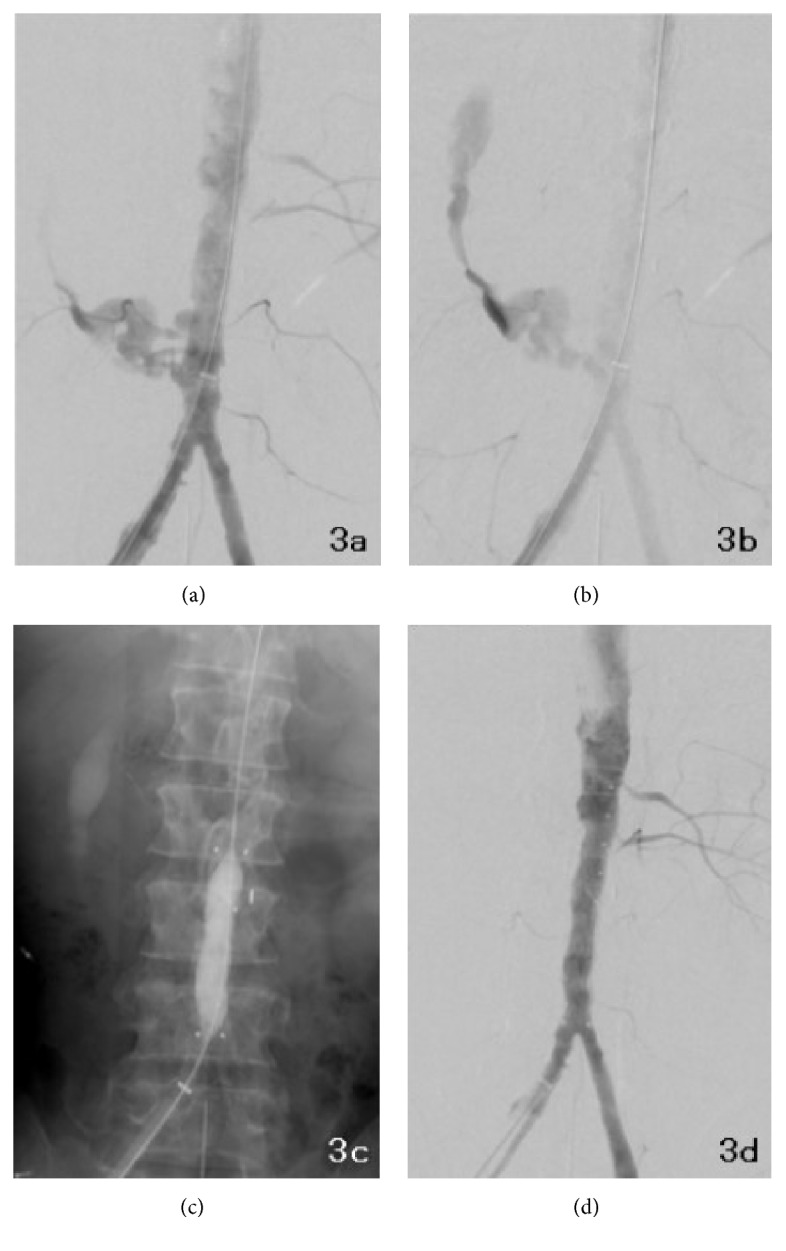
Perioperative digital subtraction angiograms. Extravasation from the right lateral wall of the abdominal aorta (a) and diffusion of the contrast agent along the duodenum (b) were observed. The celiac, superior mesenteric, and right renal arteries were occluded. A covered stent graft was successfully deployed into the abdominal aorta, and a leak at the end of the graft was treated with balloon angioplasty (c). After placement of the endovascular stent graft, aortography showed no extravasation from the right lateral wall of the abdominal aorta (d).

A 56-year-old man with a recent history of duodenal ulcer was admitted with severe hematemesis and hemorrhagic shock. On arrival, the patient presented with hematemesis and mild epigastric pain. There was no sign of sepsis including fever, chills, rigors, and highly elevated inflammatory markers. Laboratory findings showed low hemoglobin level (8.3 g/dL), mildly elevated leukocyte count (11600/dL), and low C-reactive protein level (0.3 mg/dL). The patient's respiration was unstable and chest radiography revealed bilateral aspiration pneumonia; immediate endotracheal intubation was performed. The patient had a history of orchiectomy for seminoma followed by radiation therapy for para-aortic lymph node metastases approximately 30 years before the events described herein. Additionally, the patient had undergone several open surgeries for small bowel obstruction related to radiation-induced enteritis and adhesions. Although an ulcer and stenosis of the third portion of the duodenum had been revealed one month before admission, emergency gastroduodenal endoscopy on admission failed to reveal the source of bleeding due to the massive amount of fresh blood in the stomach and duodenum. Moreover, contrast-enhanced computed tomography (CT) identified no extravasation around the duodenum, but it revealed mucosal thickening of the duodenal wall, duodenal stenosis at the level of the third and fourth portion of the duodenum, and soft-tissue density with fat stranding between the duodenum and the abdominal aorta (Figure 1). Contrast CT on admission also revealed the presence of diffuse calcification and stenosis from the left common iliac artery to the abdominal aorta, which was confined to the radiation field (Figure 2). Because these findings were suggestive of ADF, emergency aortography was performed, revealing occlusion of the origin of the celiac and superior mesenteric arteries, which were supplied via the collateral circulation. Furthermore, extravasation from the right lateral wall of the abdominal aorta and diffusion of the contrast agent along the duodenum were also observed (Figure 3), which suggested that the source of bleeding was a direct communication between the abdominal aorta and the duodenum.

A covered stent graft was deployed under fluoroscopic guidance via cannulation of the right femoral artery. The diameter of the infrarenal abdominal aorta was 12 mm, but it was only 11 mm at the narrowest site. Because it was predicted that the advancement of the aortic wall would be poor due to a high degree of arteriosclerotic change, we chose a covered stent graft (Endurant II iliac extension; width, 13 mm; length, 82 mm; Medtronic Inc., Minneapolis, MN) that was slightly bigger than the diameter of the aorta among the immediately usable devices. For adequate sealing of the stent graft, ballooning of the proximal and distal neck was performed. There were no intraoperative complications and angiography showed good positioning of the stent graft and complete exclusion of the extravasation. The ADF was completely sealed with the covered stent graft (Figure 3(d)).

After the procedure, the patient received intravenous broad-spectrum antibiotics for treatment of aspiration pneumonia and to prevent infection from ADF throughout hospital stay. Hematemesis did not recur and anemia gradually improved. Although the patient's condition temporarily recovered enough to allow rehabilitation, he died 33 days after the procedure as a result of acute exacerbation of pneumonia.

## 3. Discussion

AEF is a direct communication between the abdominal aorta and the gastrointestinal tract and represents a rare but critical cause of repeated and massive gastrointestinal bleeding. Since the first description of primary ADF was published by Astley Cooper in 1839, less than 200 primary aortoenteric fistula (AEF) cases have been reported in the English-language literature; 73% of primary AEF were from atherosclerotic aneurysms, 26% were from traumatic or mycotic aneurysms, and the remaining 1% were caused by radiation, metastases, pancreatic carcinoma, ulcers, gallstones, diverticulitis, appendicitis, and cystic medial necrosis [1]. As described in the Introduction, primary ADF involving a normal-size aorta is rare [2]; furthermore, ADF related to radiation therapy is extremely rare. Puccio reviewed the research on this uncommon condition and reported that radiation related AEF was not associated with aneurysm in all eight cases, including their own case [3]. ADF typically occurs between the third and fourth portions of the duodenum (70%–90%) [4]. Initial bleeding is usually minor and often intermittent in approximately two-thirds of cases, but this small amount of bleeding can lead to severe bleeding and hemorrhagic shock (herald bleeding) [5–7], resulting in significant mortality of up to 40%–70% [5]. The period from herald bleeding to massive bleeding ranges from hours to months.

Several reports have mentioned acute or chronic radiation-induced damage to tissues as a potential cause for ADF and suggested that minor, but sustained, damage to either of or both the duodenal wall and aortic wall can result in tissue necrosis and perforation of these walls [3, 8–12]. Histological changes related to radiotherapy include mucosal and submucosal damage due to obliteration of small vessels, telangiectasia, and new vessel formation, resulting in tissue damage and subsequent formation of fibrosis and chronic erosion (ulcer). Therefore, several authors have hypothesized that a chronic ulcer slowly erodes through the duodenal wall and eventually perforates a weakened aortic wall [3, 8]. Radiation-induced tissue damage also accelerates arteriosclerosis and aortopathy. Peripheral fibrosis, calcification, thrombosis, and severe stenosis or even occlusion of small blood vessels including the vasa vasorum induce fragility of large vessels, eventually resulting in perforation of the aorta [3, 8–12]. ADF can develop years or decades after radiation therapy [3]. As described above, the fragility of both the duodenum and aorta due to chronic radiation-induced histological damage is considered the cause of ADF in our patient, who had a history of severe enteritis and intestinal adhesions that occurred after radiation therapy. Moreover, the presence of duodenal stenosis at the level of the third and fourth portion of the duodenum, identified on contrast CT, indicated chronic inflammation and secondary fibrosis. Thus, it was estimated that the ulcer observed in the third portion of the duodenum one month before admission was also associated with radiation-induced complications. Since it is known that chronic inflammation may lead to erosion in the surrounding tissue, the CT findings of soft-tissue density with fat stranding between the duodenum and aorta suggested the presence of chronic inflammation between the duodenum and aorta. Furthermore, our patient also had diffuse calcification and stenosis confined to the radiation field, which also corresponded to findings indicative of radiation-induced arteriosclerosis or aortopathy. Hence, we believe that, in our patient, ADF was caused by radiation therapy.

Although CT typically has high diagnostic ability (sensitivity, 93%; specificity, 85.7%) for ADF formation [13], intermittent bleeding may be undetectable on contrast-enhanced CT, as it was in our patient. If ADF is suspected, angiography should be performed.

Despite prompt surgical repair, open surgeries including extra-anatomic bypass, aortic ligation, and graft replacement or open intestinal repair are associated with high mortality (27% to 50%) [13–16] and morbidity rates (66% to 80%) [13, 15, 16]. Furthermore, operative mortality rates are higher in patients with sepsis or shock [13, 15]. With respect to the optimal treatment method in the initial management of patients with severe hemorrhagic shock, endovascular aortic repair is preferred to open surgery because it is faster and less invasive, allowing rapid control of blood loss in patients with hemodynamic instability. Some researchers reported that, compared to those in laparotomy, the use of an endovascular stent graft seemed to be associated with decreased perioperative mortality and shorter hospital stay, allowing for acceptable survival given the presence of coexisting medical comorbidities [12–14, 17]; however, the mortality rate is worse for patients with sepsis [13, 14]. The reported perioperative morbidity rates of endovascular repair are lower than that of open surgery and range from 25% to 60% [13–15, 17].

In our patient, mild leukocytosis was observed before the procedure, which was most likely caused by aspiration pneumonia. However, there were no signs of sepsis preoperatively. Immediate control of bleeding was necessary, and thus endovascular repair with a stent graft seemed to be a safer option than open surgery because of the patients' unstable hemodynamic state and the presence of intestinal adhesions.

The remaining aortoduodenal communication may serve as a nidus for continuing bacterial growth and lead to infection of the new implanted endovascular stent graft; therefore, long-term intravenous antibiotic therapy is necessary. Baril et al. [13] reported that all patients treated with endovascular repair were placed on lifelong suppressive oral antibiotics, which may allow for freedom from further complications related to ADF. Postoperative stent graft infection and recurrent bleeding are known as the most frequent causes of death; such an event is significantly more frequent in patients with sepsis before endovascular treatment. However, even if patients have ongoing sepsis or other infectious complications, endovascular repair may serve as a bridging therapy to open surgery [13–15, 17]. In our case, laparotomy was expected to be difficult due to severe adhesions; however, a variety of adjunctive therapies including fibrin sealant or n-butyl 2-cyanoacrylate insulation into a fistula tract might be an option [18, 19].

We reported the case of ADF related to radiation-induced arteriosclerosis/aortopathy and enteropathy that was successfully sealed with an endovascular stent graft. While ADF related to radiotherapy is extremely rare, it may be the cause of fatal gastrointestinal bleeding in patients with a history of radiotherapy. When gastroduodenal endoscopy and CT fail to detect ADF, angiography and endovascular repair with a stent graft can be employed to rapidly diagnose and repair ADF.
